# Agro-economic and socio-environmental assessments of food and virtual water trades of Iran

**DOI:** 10.1038/s41598-021-93928-9

**Published:** 2021-07-22

**Authors:** Fatemeh Karandish, Hamideh Nouri, Marcela Brugnach

**Affiliations:** 1 Water Engineering Department, University of Zabol, Zabol, Iran; 2 Multidisciplinary Water Management, Faculty of Engineering Technology, University of Twente, Enschede, The Netherlands; 3 Division of Agronomy, University of Göttingen, Von-Siebold-Strasse 8, 37075 Göttingen, Germany; 4 Basque Centre for Climate Change, Scientific Campus of the University of the Basque Country, 48940 Leioa, Spain; 5 Basque Foundation for Science, Ikerbasque, Bilbao, Spain

**Keywords:** Environmental social sciences, Hydrology, Climate sciences

## Abstract

Ending hunger and ensuring food security are among targets of 2030’s SDGs. While food trade and the embedded (virtual) water (VW) may improve food availability and accessibility for more people all year round, the sustainability and efficiency of food and VW trade needs to be revisited. In this research, we assess the sustainability and efficiency of food and VW trades under two food security scenarios for Iran, a country suffering from an escalating water crisis. These scenarios are (1) Individual Crop Food Security (ICFS), which restricts calorie fulfillment from individual crops and (2) Crop Category Food Security (CCFS), which promotes “eating local” by suggesting food substitution within the crop category. To this end, we simulate the water footprint and VW trades of 27 major crops, within 8 crop categories, in 30 provinces of Iran (2005–2015). We investigate the impacts of these two scenarios on (a) provincial food security (FS_p_) and exports; (b) sustainable and efficient blue water consumption, and (c) blue VW export. We then test the correlation between agro-economic and socio-environmental indicators and provincial food security. Our results show that most provinces were threatened by unsustainable and inefficient blue water consumption for crop production, particularly in the summertime. This water mismanagement results in 14.41 and 8.45 billion m^3^ y^−1^ unsustainable and inefficient blue VW exports under ICFS. “Eating local” improves the FS_p_ value by up to 210% which lessens the unsustainable and inefficient blue VW export from hotspots. As illustrated in the graphical abstract, the FS_p_ value strongly correlates with different agro-economic and socio-environmental indicators, but in different ways. Our findings promote “eating local” besides improving agro-economic and socio-environmental conditions to take transformative steps toward eradicating food insecurity not only in Iran but also in other countries facing water limitations.

## Introduction

Food security is a global challenge under an ever-increasing population and ever-shrinking finite resources. Currently, about one billion (16%) of the world’s population are undernourished, 80% of which live in the global south^1^. Globally, fulfilling food demand is facing two main challenges concerning land and water availability^2,3^. On one hand, global croplands are significantly reduced by urbanization growth, the overexploitation of fertile lands and deforestation^4^. On the other hand, food production within the remaining croplands is also restricted by ever-shrinking finite water resources. This is resulting in an intensification of management strategies, such as improving agricultural practices or water management to enhance the productivity of cropping systems^5^. However, the food security achieved under these strategies is, in some nations, mainly at the expense of the environment; in particular of freshwater resources^3^. These strategies cannot last long, unless they are systematically revisited based on the availability and sustainability of resources^1,6^.

Among numerous quantitative indicators, water footprint (WF)^7^ is known as a multidimensional indicator of water consumption, which can be used to formulate pathways to achieve sustainable water resource development in agriculture. Along with WF assessment (WFA), recent studies proved that water scarcity could be alleviated through efficient water use in croplands by mulching and efficient irrigation systems^8,9^, changing cropping patterns^9^, or benchmarking^10,11^. Virtual water (VW) trade is another concept that was developed to monitor and manage embedded water in crop trades. Water-smart food trade in water-scarce regions, i.e. importing water-intensive crops instead of domestic production, has the potential to achieve higher levels of food and water security compared to localized food production from a water management point of view^12,13^. On the contrary, water scarcity in exporting regions could be escalated when VW flows are issued in the opposite direction^14,15^.

Many countries in the world are involved in VW trade and satisfy their food demand through the import/export process. Additionally, increasing water trades become problematic since they, not only decrease already scarce water resources, but also reduce the resilience of communities toward unanticipated crises^16^. Indeed, a VW trade is labeled sustainable when the products are not produced at and exported from hotspots, where the water consumption exceeds the sustainable availability of water. Crop-related VW trade is efficient when the WFs of the produced crops in the exporting regions are lower than their benchmark levels. The literature review reveals that little research has been done on the sustainability and efficiency of inter- or intra-national VW trade. In Iran, relocating wheat cultivation may modify crop and VW trade direction to reduce consumption in water-scarce provinces^17^. In the UK, for example, it was claimed that about 50% of the imported VW was unsustainable and inefficient since it originated from countries with blue water scarcity (BWS) > 1 and WF values beyond the benchmark levels^18^. Another study revealed that cotton consumption in Germany exacerbates water scarcity in Pakistan, and deteriorates the quality of drinking water. Hence, a global effort should raise awareness on the impacts of externalizing the water consumption of a nation and its repercussions on water resources of another nation^19^. A crop-related WF assessment of Iran demonstrated that the direction of provincial VW trade occurred from the water-scarce provinces to the water-abundant ones^20^.

Here, and for the first time, we assessed the efficiency and sustainability of the inter-provincial crop-related VW export under different food security scenarios as a case study for Iran, the second largest country in the most water scarce region of the world, The Middle East and North Africa (MENA)^21^. Iran is facing a severe BWS, and irrigated agriculture contributes to 97% in the total net blue water abstraction^13,22^. Agriculture plays a key role in Iran’s economy with a contribution of 13% in the national gross domestic production, 20% in employment rate, 23% in the non-oil export, 82% in total food consumed by the residents, and 90% in total raw materials used by the food-processing factories^17^. In 1979, self-sufficiency in food production was implemented in the center of Iran’s food policies which promoted national self-sufficiency in terms of agricultural production, mainly for cereal production^20^. Nevertheless, the national self-sufficiency plan has not yet been achieved and Iran relies on other countries' water bodies to meet its food demand^20^. Due to the substantial contribution of agriculture in the national water consumption, we assessed the potentials for improving crop-related blue water consumption and trade in the hopes of getting sustainable self-sufficient food security in Iran while reducing water-related challenges.

In this study, we first evaluated the provincial food security levels from domestic agricultural production; the influence of current production patterns on the efficiency and sustainability of blue water consumption and VW trade. Thereafter, we tested the relationship between efficiency/suitability and self-sufficient food security levels; and suggested potentials for improving the current conditions. These potentials were extracted from agro-economic and socio-environmental assessments using different agricultural, economic, environmental, and social indicators.

## Results

### Food security and export

Table 1 shows the FS_p_ values obtained from each crop category and from all 27 crops, under ICFS and CCFS scenarios. Considering all 27 selected crops, the overall FS_p_ value varies in the range of 11–98% and 16–100% under ICFS and CCFS scenarios, respectively. Indeed, considering crop substitution within a specific crop category increases the provincial FS_p_ values by 1–210%.

**Table 1:** Provincial level of self-supplied crop-related food security under ICFS and CCFS scenarios.

Province	Self-supplied food security level (%) under ICFS scenario^a^									Self-supplied food security level (%) under CCFS scenario								
	Cereals	Root and tubers	Sugar crops	Pulses	Nuts	Oil crops	Vegetables	Fruits	All crops^b^	Cereals	Root and tubers	Sugar crops	Pulses	Nuts	Oil crops	Vegetables	Fruits	All crops
Ardebil	82	38	100	58	25	9	52	17	76	100	38	100	84	25	9	56	40	92
AzarGharbi	82	78	69	6	42	28	41	41	76	100	78	69	6	90	28	41	100	94
AzarSharghi	83	82	100	4	6	70	100	41	81	100	100	100	4	6	70	100	100	99
Bushehr	41	83	4	4	0	100	0	70	44	50	100	4	4	0	100	0	100	54
Chaharmahal	88	6	100	16	33	100	100	41	83	100	6	100	16	33	100	100	85	96
Esfahan	77	68	100	6	42	60	0	24	71	100	100	100	6	46	60	0	32	90
Fars	99	100	91	19	42	100	100	88	98	100	100	91	32	75	100	100	100	100
Ghazvin	85	69	77	12	42	100	46	41	80	100	100	77	12	51	100	46	100	97
Ghom	18	2	1	6	0	100	7	10	20	39	2	1	6	0	100	7	10	36
Guilan	24	10	8	2	0	54	100	17	26	100	10	8	2	0	54	100	17	81
Gorgan	100	67	100	100	0	0	26	18	86	100	100	100	100	0	0	26	20	87
Hamedan	82	69	100	18	42	100	100	41	80	100	69	100	37	52	100	100	100	99
Hormozgan	16	100	56	1	0	51	1	49	23	30	100	56	1	0	51	1	100	37
Ilam	88	49	4	9	2	49	10	9	75	100	49	4	9	2	49	11	9	85
Kerman	57	33	100	1	13	100	16	65	59	94	33	100	1	13	100	16	100	92
Kermanshah	82	47	15	6	42	100	39	40	75	100	47	15	6	42	100	39	72	91
Khuzestan	96	100	26	5	78	9	62	37	82	100	100	26	5	100	9	62	77	88
Kohgiluieh	83	28	1	18	10	39	76	52	67	100	28	1	23	10	39	77	100	83
Kordestan	82	29	100	6	1	100	19	37	78	100	29	100	6	1	100	19	45	92
Lorestan	86	71	100	23	26	100	100	40	84	100	100	100	23	26	100	100	47	97
Markazi	81	81	100	19	16	100	100	41	81	100	100	100	24	16	100	100	100	100
Mazandaran	42	25	26	100	1	16	32	19	39	100	25	26	100	1	16	32	100	90
North-Khorasan	82	100	100	7	42	67	70	41	80	100	100	100	7	100	67	71	100	98
Razavi-Khorasan	50	75	22	3	0	100	9	41	50	73	100	22	3	0	100	9	100	72
Semnan	81	91	100	4	42	100	100	34	80	100	100	100	6	56	100	100	100	100
Sistan	42	59	10	1	0	100	0	79	42	53	82	10	1	0	100	0	100	53
South-Khorasan	82	100	100	6	42	100	100	49	82	100	100	100	6	100	100	100	49	97
Old-Tehran	10	25	16	1	2	13	7	13	11	16	25	16	1	2	13	7	13	16
Yazd	52	64	15	1	3	100	18	45	52	76	64	15	1	3	100	18	63	72
Zanjan	86	100	100	12	40	100	100	41	85	100	100	100	12	40	100	100	100	100

ICFS is Individual Crop Food Security, which restricts calorie fulfillment from individual crops and CCFS is Crop Category Food Security, which promotes “eating local” by suggesting food substitution within the crop category.

^a^For a specific crop category, a province with a food security level of 100% is entirely self-sufficient in supplying domestic demand; and could be a food exporter to provinces with deficit production.

^b^A province could have an overall food security level of < 100%, although being food exporter for some specific crop categories.

For both scenarios, the overall FS_p_ value strongly correlates with cereal’s FS_p_ values, where provinces with higher contributions in cereal production have higher FS_p_ values. Irrigated agriculture has a crucial role in satisfying food security in Iran since roughly 66–100% of the overall FS_p_ value is supplied by the irrigated crops (Table 2).

**Table 2:** Total and unit irrigated-crop, blue VW export, and unsustainable blue VW export for different crop categories under the ICFS and CCFS scenarios over the study period (2005–2015).

Crop category	ICFS scenario						CCFS scenario					
	Crop export		Blue VW export				Crop export		Blue VW export			
	Total export	Unit export	Total export	Unit export	Unsustainable export	Unit unsustainable export	Total export	Unit export	Total export	Unit export	Unsustainable export	Unit unsustainable export
	(10^6^ t y^−1^)	(t ha^−1^)	(10^9^ m^3^ y^−1^)	(m^3^ ha^−1^)	(10^9^ m^3^ y^−1^)	(m^3^ ha^−1^)	(10^6^ t y^−1^)	(t ha^−1^)	(10^9^ m^3^ y^−1^)	(m^3^ ha^−1^)	(10^9^ m^3^ y^−1^)	(m^3^ ha^−1^)
Cereals	9.51	1.82	12.21	2343	6.18	1185	7.41	1.42	9.75	1872	5.19	996
Root and tuber	2.56	12.31	0.39	1891	0.21	1006	2.56	12.31	0.39	1891	0.21	1006
Sugar crops	7.77	35.36	2.12	9655	1.61	7329	7.03	32.00	1.89	8591	1.50	6844
Pulses	0.28	0.63	1.49	3352	0.66	1472	0.20	0.45	0.98	2197	0.32	721
Nuts	0.56	1.00	3.08	5543	1.83	3301	0.56	1.00	3.08	5543	1.83	3301
Oil crops	0.33	1.15	0.65	2267	0.45	1588	0.26	0.93	0.51	1776	0.36	1268
Vegetables	3.63	13.56	1.11	4141	0.74	2772	3.21	12.00	1.00	3715	0.68	2533
Fruits	5.36	7.73	5.26	7585	2.73	3942	3.60	5.19	3.87	5584	2.09	3011
All crops	30.00	3.80	26.32	3337	14.41	1828	24.84	3.15	21.47	2722	12.19	1546

Under the ICFS scenario, a total of 30.0 million tons of irrigated crops are inter-provincially traded per year; cereals and sugar crops have the highest contribution in this export. Under the CCFS scenario, the inter-provincial crop trade decreases 17% compared to the ICFS scenario; noticeably for cereals and fruits.

### Sustainability and efficiency of blue water consumption

**Figure 1:**
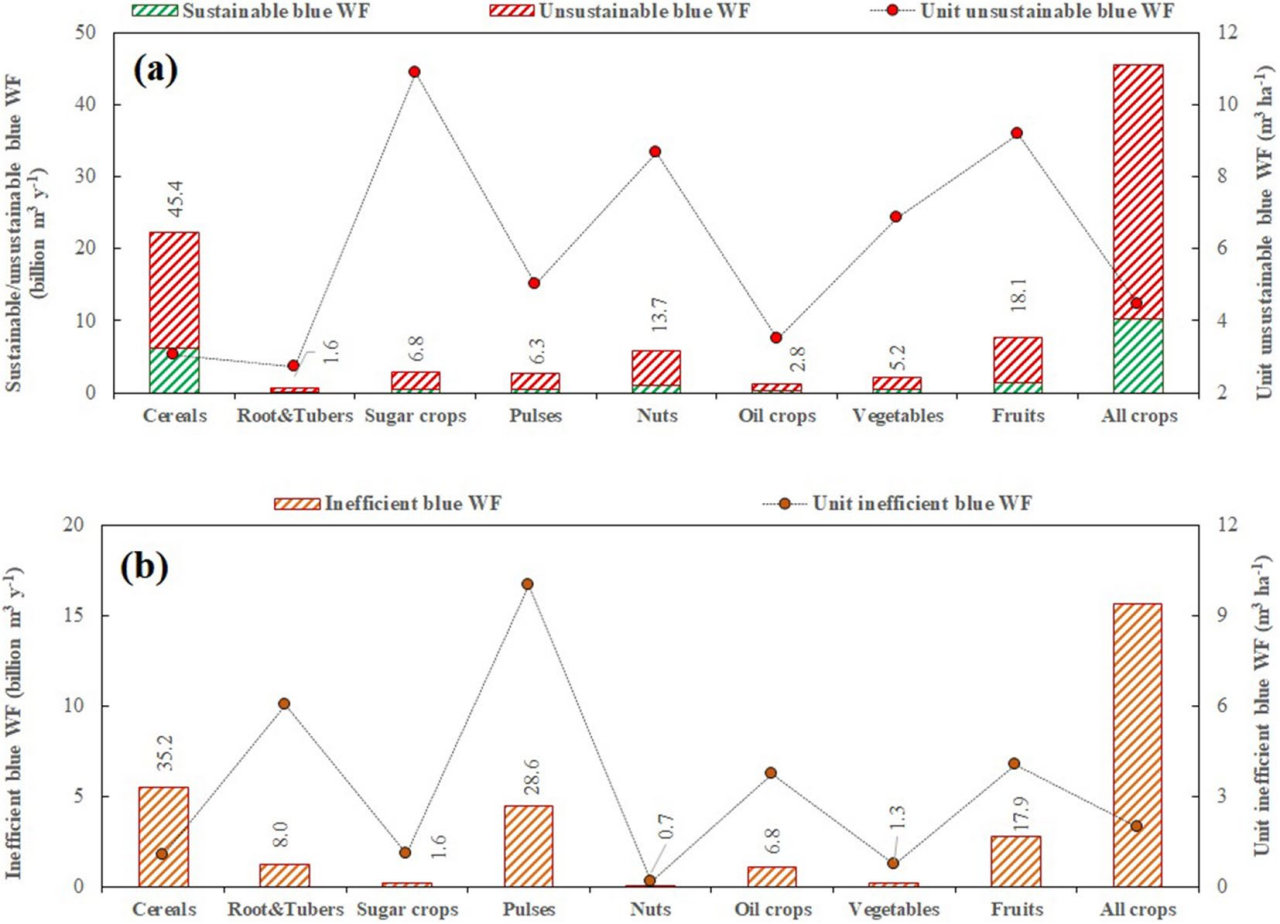
Annual total (m^3^ y^−1^) and unit (m^3^ ha^−1^) (**a**) sustainable/unsustainable and (**b**) inefficient blue WFs for different crop categories Iran over the study period (2005–2015). The unit unsustainable or inefficient blue WFs (m^3^ ha^−1^) was estimated by dividing total unsustainable or inefficient blue WFs (m^3^ y^−1^) by the area of croplands (ha) in the specific province. (i.e., The Figure is created in the environment of Microsoft Office—Excel—version 10.).

On the annual scale, 45.5 billion m^3^ of blue water is consumed to produce these 27 major crops, 78% (35.5 billion m^3^ y^−1^) of which is unsustainable consumption, and 34% of which (15.5 billion m^3^ y^−1^) is inefficient consumption. Figure 1 shows that cereals are major contributors in both the annual unsustainable and inefficient blue water consumption (m^3^ y^−1^, 46% and 35%, respectively). However, by unit measurements of unsustainability and inefficiency (m^3^ ha^−1^) indicate that sugar crops have the highest unsustainable blue WF value (10.9 thousand m^3^ ha^1^) and that pulses have the highest inefficiency (thousand 10.0 m^3^ ha^−1^).

**Figure 2:**
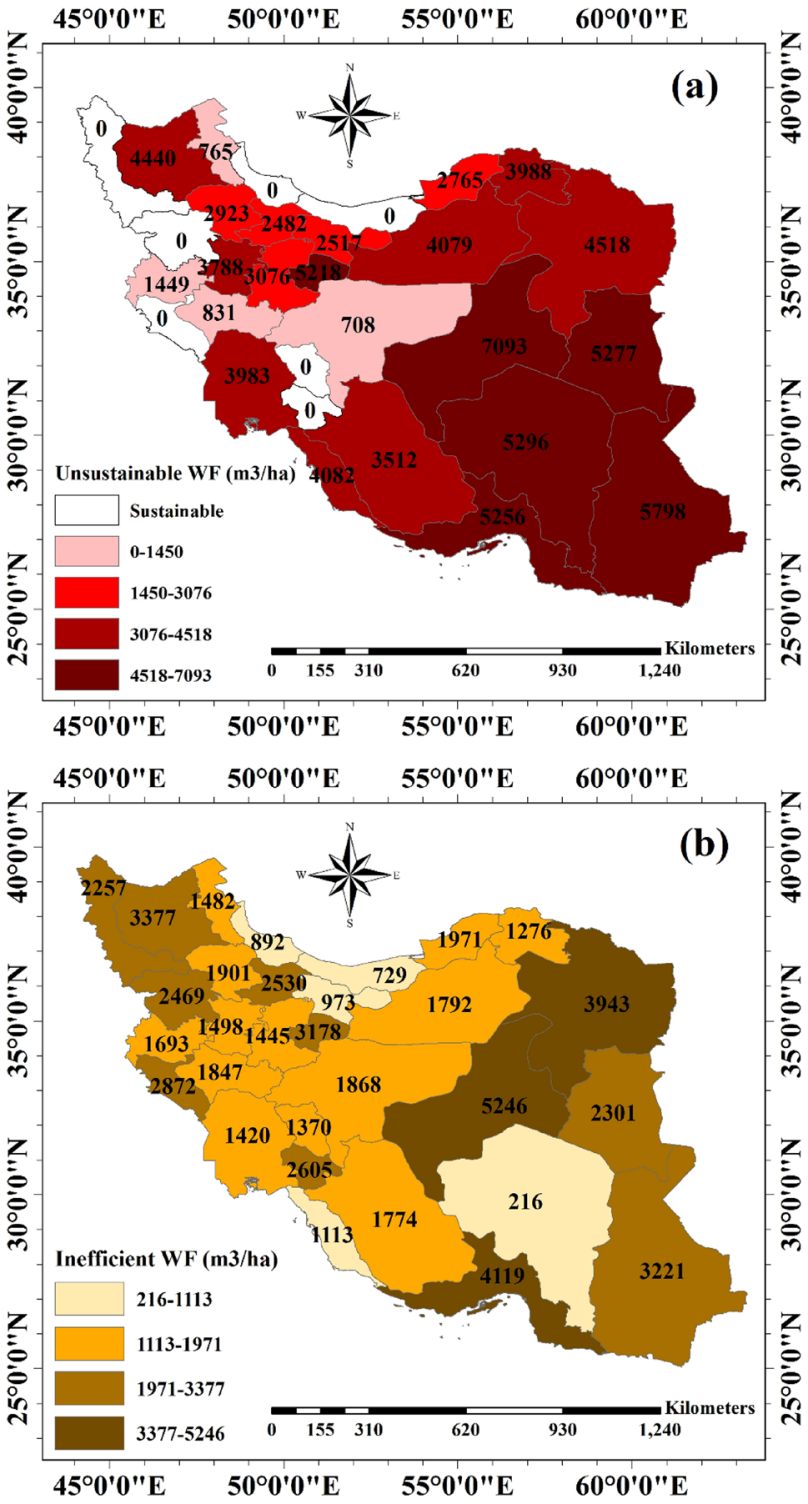
Provincial unit (**a**) unsustainable and (**b**) inefficient blue water footprint (WF) per unit of cropland (m^3^ ha^−1^) over the study period (2005–2015). Per province, unit unsustainable or inefficient blue WFs was calculated by dividing the total unsustainable or inefficient blue WFs (m^3^ y^−1^) by the area of croplands (ha) in the specific province. (i.e., This Figure is created in the environment of ArcMap-GIS version 10.7).

Provincial unit unsustainable blue WF values (m^3^ ha^−1^) vary from 0.71 thousand m^3^ ha^−1^ (Esfahan) to 7.1 thousand m^3^ ha^−1^ (Yazd) in hotspot provinces depending on their cropping pattern, accessibility to different sources, and agricultural practices and water management (Fig. 2). In seven provinces, blue water consumption is sustainable due to high blue water availability. There is no province in which blue water consumption is fully efficient. Unit inefficient blue WF values vary from 0.2 thousand m^3^ ha^−1^ (Kerman) to 5.3 thousand m^3^ ha^−1^ (Yazd).

### Sustainability and efficiency of blue VW export

#### Inter-provincial sustainability of blue VW export under the ICFS and CCFS scenarios

**Figure 3:**
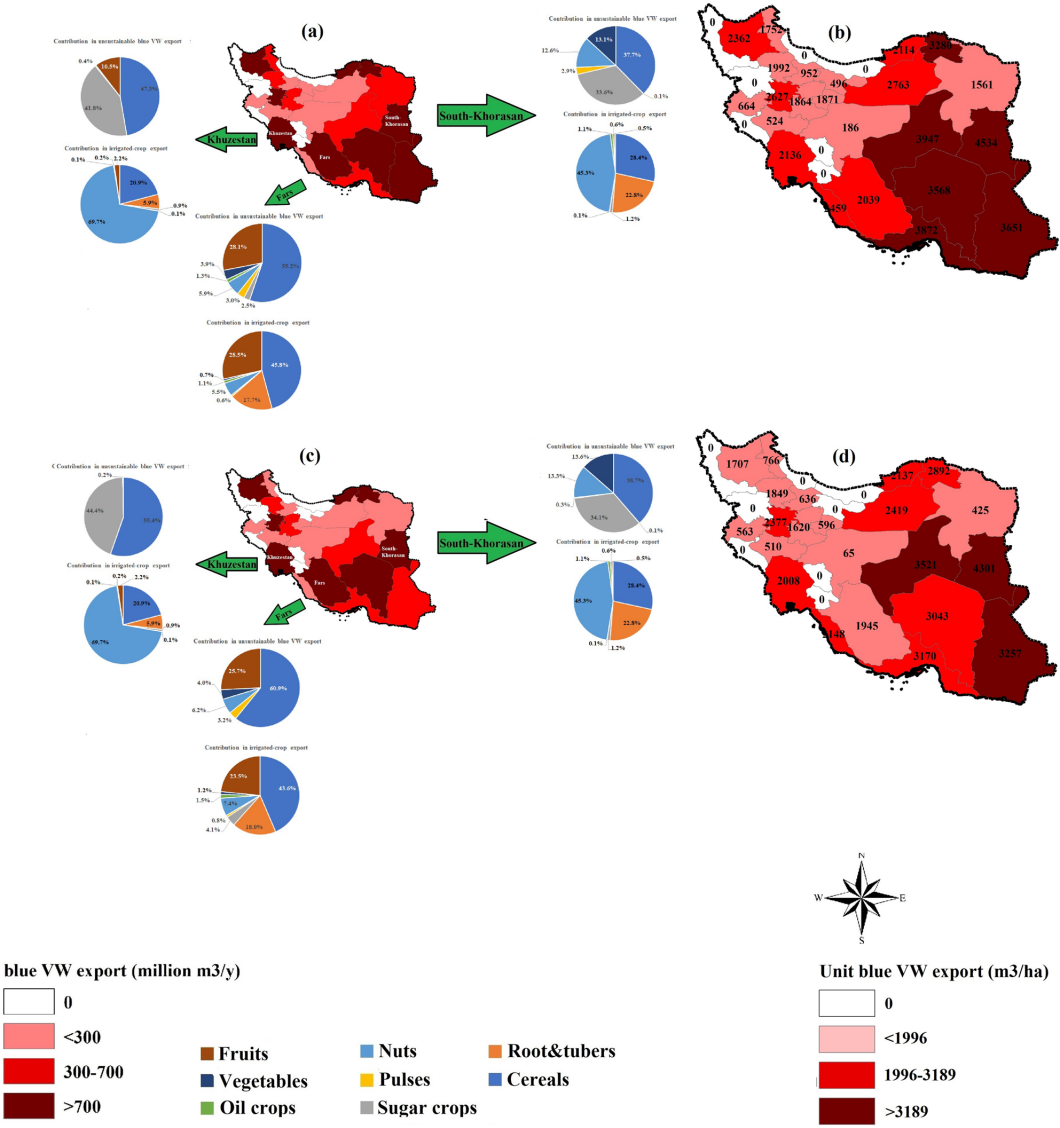
Inter-provincial values of the total (**a** and **c**) and unit (**b** and **d**) unsustainable blue virtual water (VW) exports under ICFS (left) and CCFS (right) scenarios. The contribution of eight crop categories in the total irrigated crop export and total unsustainable blue VW export are reported in top-three hotspots of—Fars, Khuzestan, and South-Khorasan provinces. Per province, unit unsustainable blue VW exports (m^3^ ha^−1^) were estimated by dividing the total unsustainable blue VW export (m^3^ y^−1^) by the area of croplands (ha) in the specific province. (i.e., This Figure is created in the environment of ArcMap-GIS version 10.7).

*ICFS scenario*—23 out of 30 provinces have unsustainable blue VW export; accounting for 0.59 billion m^3^ y^−1^ to 1.85 billion m^3^ y^−1^ (Fig. 3a). Three provinces of Fars, Khuzestan and South-Khorasan, three major crop-growing areas of Iran with a contribution of 12%, 18% and 5% in the national production, have the greatest unsustainable blue VW export; accounting for 1.85, 1.61 and 1.57 billion m^3^ y^−1^, respectively. These provinces contribute the most in the inter-provincial irrigated-crop export (11%, 21% and 7%, respectively) while they are classified as severe blue water scarce regions based on the BWS index.

The exported crop type from a hotspot (i.e., a region with BWS > 1) affects the absolute unsustainable blue VW export. Fars province has a lower contribution to the total inter-provincial crop export (11%) than the Khuzestan province (21%), but its contribution in total unsustainable blue VW export is larger. In the Fars province, cereals and fruits are major exports and both have higher unsustainable blue WF values compared to sugar crops (Table 2), which are major exports in the Khuzestan province. South-Khorasan province, however, has a lower unsustainable blue VW export value because of its smaller contribution in the total inter-provincial crop export (7%).

In sustainability assessment from the angle of blue VW export per unit of cropland (m^3^ ha^−1^), South-Khorasan is the most critical province among thirty provinces of Iran (Fig. 3b). It has the highest unit unsustainable blue WF value (4.53 thousand m^3^ ha^−1^), which is 122% and 112% higher than those in the provinces of Fars and Khuzestan. This is why the BWS value in South-Khorasan (9.1) is noticeably higher than those in Khuzestan (3.6) and Fars (2.2). Therefore, the fraction of unsustainable blue WF value for a specific crop in South-Khorasan is larger than the same crop in Fars or Khuzestan. Hence, South-Khorasan is the most vulnerable province to environmental deterioration and agricultural drought, and crop exports from this province is not justified from an environmental point of view.

*CCFS scenario—*the number of provinces with unsustainable blue VW export decreases from 23 to 22 provinces (Fig. 3c). This one different province is Old-Tehran, which includes New-Tehran as the capital of Iran with the largest population and rate of urbanization, but the smallest contribution in national croplands. As expected, the New-Tehran province consumes its domestic own crop production with little left to export. No export from this province leads to zero unsustainable blue VW export value. In other provinces, the inter-provincial unsustainable blue VW export varies from 0.02 billion m^3^ y^−1^ (Esfahan) to 1.76 million m^3^ y^−1^ (Fars). Nevertheless, the hotspot provinces remain unchanged; Fars, Khuzestan and South-Khorasan. These provinces have the largest contributions in the total unsustainable blue VW exports; accounting for 1.87 billion m^3^ y^−1^, 1.69 billion m^3^ y^−1^, and 1.46 billion m^3^ y^−1^, respectively. In addition, South-Khorasan has the highest unit of unsustainable blue VW export; although its value decreased by 5.1% to 4.3 thousand m^3^ ha^−1^ compared to the one under ICFS scenario (Fig. 3d).

The total and unit unsustainable blue VW export, for different crop categories between ICFS and CCFS scenarios were compared over the period 2005–2015 (Table 2).

Under ICFS scenario, a total of 26.3 billion m^3^ y^−1^ of blue VW is interprovincially exported; 55% (14.4 billion m^3^ y^−1^) of which is unsustainable. Cereals have the highest contribution (43%), followed by fruits (19%). Such high cereal production rates are due to its dominant contribution to the national croplands and productions (see Fig. S2 in the supplementary information). However, when unit unsustainable blue VW exports (m^3^ ha^−1^) are considered, producing sugar crops, fruits and nuts become less sustainable (Table 2).

Under the CCFS scenario, the total blue VW export decreases by 18% (21.5 billion m^3^ y^−1^), which in turn results in an 17% reduction in the total irrigated-crops export. Consequently, the total unsustainable blue VW export decreases by 15% (12.2 billion m^3^ y^−1^). Crop substitution in food diets also reduces crop-category-specific unsustainable blue VW export by 7% (in sugar crops) to 51% (in pulses). Similarly than in the ICFS scenario, cereals and fruits have major contributions in the overall inter-provincial unsustainable blue VW export, with 43% and 17% respectively. Unit unsustainable blue VW exports also decrease up to 51% while the critical crops remain the same as the values under the ICFS scenario.

#### Inter-provincial efficiency of blue VW export under the ICFS and CCFS scenarios

**Figure 4:**
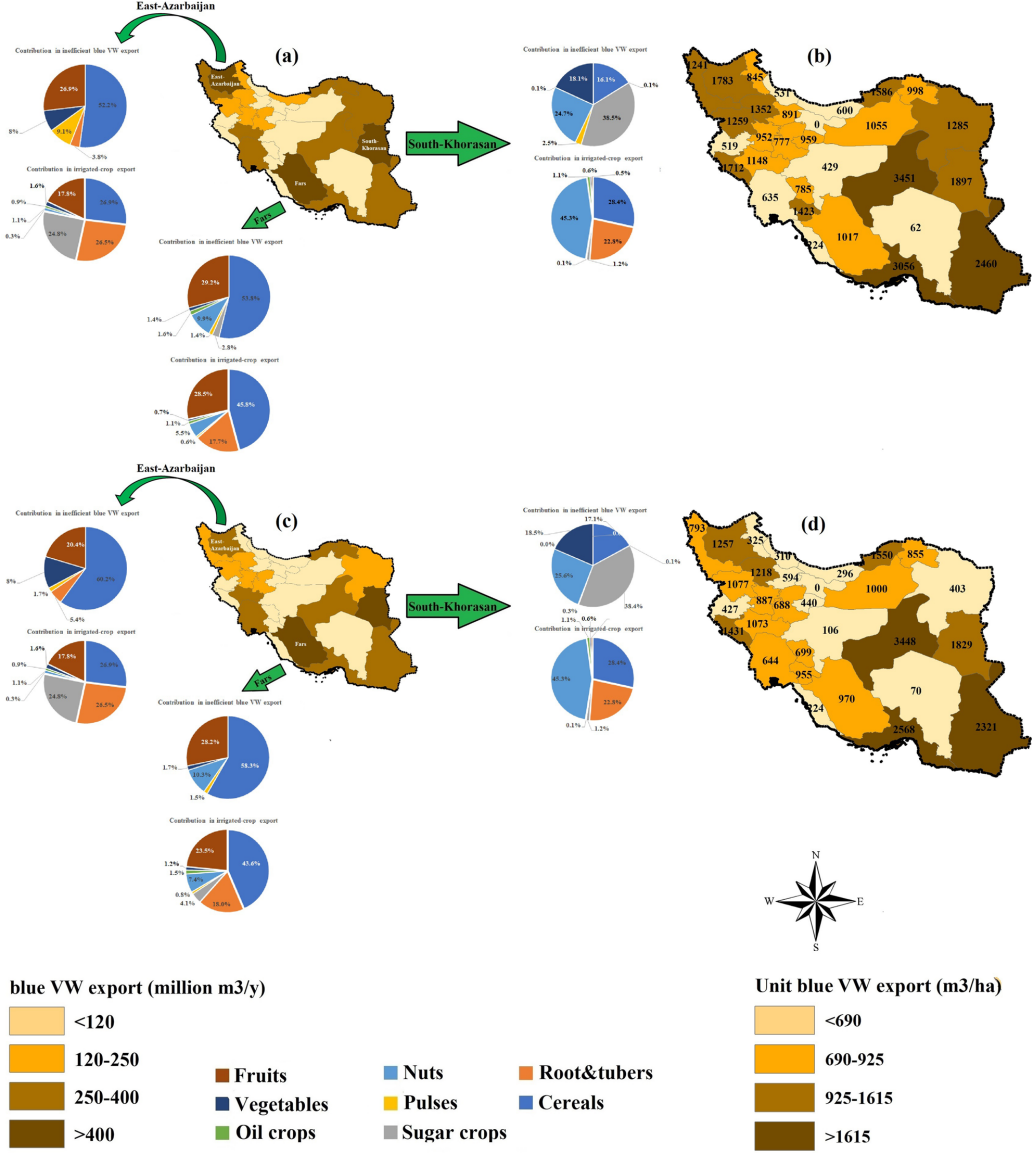
Inter-provincial values of the total (**a** and **c**) and unit (**b** and **d**) inefficient blue virtual water (VW) exports under ICFS (left) and CCFS (right) scenarios. The contribution of eight crop categories in the total irrigated crop export and total inefficient blue VW export are reported in top-three hotspots of Fars, Khuzestan, and South-Khorasan provinces. Per province, unit inefficient blue VW exports (m^3^ ha^−1^) were estimated by dividing the total unsustainable blue VW export (m^3^ y^−1^) by the area of croplands (ha) in the specific province. (i.e., This Figure is created in the environment of ArcMap-GIS version 10.7).

*Under the ICFS scenario*, except for Old-Tehran, the contribution of provinces to inefficient export varies from 0.18 billion m^3^ y^−1^ (Bushehr) to 0.92 billion m^3^ y^−1^ (Fars), as presented in Fig. 4. Three provinces of Fars, East-Azarbaijan, and South-Khorasan have the largest inefficient blue VW exports (11%, 9% and 8% respectively). In Fars and East-Azerbaijan provinces cereals are major contributors, while in South-Khorasan sugar crops are. The total irrigated-crop export from East-Azerbaijan (1.6 million t y^−1^) is 28% less than in the South-Khorasan province (2.2 million t y^−1^); however, its higher contribution in inefficient blue VW export is due to the export of cereals and fruits, both of which have higher inefficient blue WF values (m^3^ t^−1^) compared to sugar crops in South-Khorasan.

Regarding unit inefficient blue VW export, the values (m^3^ ha^−1^) in Yazd, Homozgan, and Sistan are critical, as these provinces export larger rates of inefficient blue water (3451 m^3^ ha^−1^, 3056 m^3^ ha^−1^, and 2460 m^3^ ha^−1^, respectively).

*Under the CCFS scenario*, the inter-provincial inefficient blue VW export value is reduced from 0.01 million m^3^ y^−1^ (Bushehr, 0.1%) to 371 million m^3^ y^−1^ (Razavi-Khorasan, 69%). Hotspot provinces remain unchanged. The unit inefficient blue VW export decreases by 0.1–75% in different provinces compared to those under the ICFS scenario.

The total and unit inefficient blue VW export, for different crop categories between ICFS and CCFS scenarios were compared over the period 2005–2015 (Table 3).

**Table 3:** Total and unit inefficient blue VW export for different crop categories under the ICFS and CCFS scenarios over the study period (2005–2015).

Crop category	ICFS scenario		CCFS scenario	
	Inefficient blue VW export	Unit inefficient blue VW export	Inefficient blue VW export	Unit inefficient blue VW export
	(10^9^ m^3^ y^−1^)	(m^3^ ha^−1^)	(10^9^ m^3^ y^−1^)	(m^3^ ha^−1^)
Cereals	3.60	691	2.96	568
Root and tuber	0.13	611	0.13	611
Sugar crops	0.58	2653	0.50	2263
Pulses	0.53	1198	0.34	761
Nuts	0.91	1633	0.91	1633
Oil crops	0.32	1121	0.26	915
Vegetables	0.39	1451	0.36	1336
Fruits	1.99	2868	1.42	2042
All crops	8.45	1071	6.86	870

*Under ICFS scenario*, a total of 8.45 billion m^3^ y^−1^ of inefficient blue VW is inter-provincially exported (32% of total), 43% of which occurred from cereal lands and 24% from fruit lands. By the unit inefficient blue VW export is expressed in terms of m^3^ ha^−1^, where sugar- and fruit crops have the highest inefficient blue VW export.

*Under CCFS scenario*, the total inefficient blue VW export decreases by 19% to 6.86 billion m^3^ y^−1^. This reduction is, in turn, caused by 8% (for vegetables) to 37% (for pulses) reductions in crop-category-specific inter-provincial inefficient blue VW exports. Nevertheless, Cereals and fruits still have the highest contributions (43% and 21% respectively). In addition, critical crops regarding their unit inefficient blue VW export value (m^3^ ha^−1^) stay the same, as verifiable in the ICFS scenario.

## Discussion

The sustainability and efficiency of crop production and VW trades under two food security scenarios of ICFS and CCFS were evaluated. Our results show that eating locally by adopting crop substitutions in food diets under the CCFS scenario improves the FS_p_ value up to 210% compared to the ICFS scenario; this leads to an 17% reduction in the inter-provincial crop trade (Table 1).

Under the CCFS scenario, localized food marketing will be structured more efficiently; which helps farmers economically prosper. When farmers establish their own farm businesses, there is no need to split their incomes with the provincial trade brokers; hence consumers can additionally receive cheaper foods. On the other hand, food availability and accessibility may increase under CCFS scenario when inter-provincial food trade is not feasible. The distance between the trading partners is always a restriction to bilateral trades. Such a barrier could become more severe during circumstances such as the COVID19 pandemic. Indeed, inter-provincial crop trades increase the resilience in handling local shock (i.e., such as drought or the other natural or unnatural hazards which cause agricultural losses), but simultaneously increase the vulnerability to market shocks. Therefore, small-scale farming and local market improvements could serve as tools for reducing food dependency and valuing individual food producers^23^. At the same time, all provinces can contribute incrementally to local food production by promoting strategies, such as improving crop/water productivities, reducing food losses, and/or changing local food diets in order to increase local food availability.

Simultaneously, the environment benefits from local eating since inter-provincial food transportation is decreased under the CCFS scenario (Table 2), for instance, by reducing energy consumption and transportation-related GHG emissions^24^. Furthermore, less food transportation is associated with less food losses^25,26^. Food loss through transportation is translated into eco-environmental resource losses including water, energy and capital losses. Reduced inter-provincial food trade under the CCFS scenario results in a 16% reduction in unsustainable and a 19% reduction in inefficient inter-provincial blue VW export value (Tables 2 and 3). Indeed, the CCFS scenario lessens the pressure of in-need provinces on the exporting regions, in particular in hotspot provinces. An elimination of this pressure allows decision makers to use finite local resources more sustainably and efficiently in order to prioritize their own local demand in a sustainable manner.

**Figure 5:**
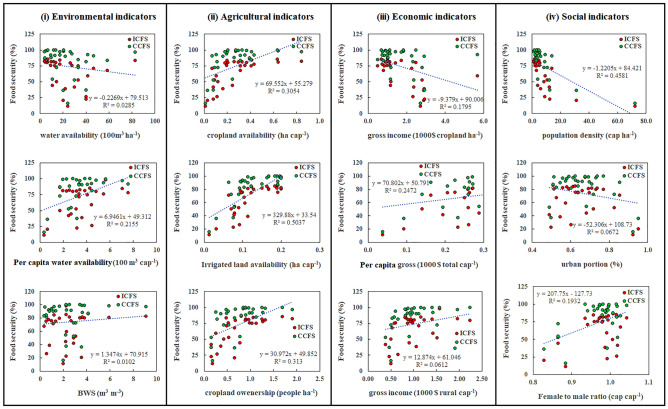
The correlations between self-supplied provincial food security and the selected (i) environmental, (ii) agricultural, (iii) economic, and (iv) social indicators. (i.e., The Figure is created in the environment of Microsoft Office—Excel—version 10.).

Adverse impacts of food trades have been fostering movements towards increasing local food supply provisions, even at a global scale^27,28^, calling for a power shift from large agricultural companies and global markets to local actors that can lead to a more sustainable crop production^23^. Furthermore, it has been acknowledged that, ensuring the sustainability and stability of food security requires a broader assessment that is not restricted to technical aspects and includes economic, social and environmental factors. Hence, we tested the relationship between different agro-economic and socio-environmental indicators and FS_P_ values (Fig. 5).

*Environmental indicators* have various effects on the local FS_p_ values. While FS_p_ values positively correlate with per capita blue water availability (m^3^ cap^−1^) (Fig. 5i), they respond adversely to any increase in water availability per unit of cropland (m^3^ ha^−1^) (Fig. 5i). In regions with higher blue water availability per unit of cropland, the contribution of cereal production (dominant crops in Iranian’ food basket) into the total production is lower; which in turn results in lower FS_p_ values._._ This explains why farmers in these regions are in favor of cash crops (e.g. fruits) rather than staple crops. This finding is in line with the correlation between FS_p_ values and BWS values (Fig. 5i); water-scarce provinces contribute more in staple crop production and have the highest FS_p_ values. The cultivation of water-intensive crops in hotspots intensifies environmental deterioration rates and threatens sustainable agriculture. Consequently, long-term food security plans necessitate an urgent revision in the cropping pattern in these regions. Effective policies need to be implemented to bridge the gap between provincial food demands and supplies while considering environmental protection.

In addition, Fig. 5 shows that reducing the portion of inefficient blue water consumption helps improve FS_p_. Several researchers demonstrated the positive effects of crop redistribution on efficient water consumption^9,29^ besides other resilient agricultural practices^30,31^. Under the condition of climate change, implementing climate-resilient pathways in the agricultural sector is a necessity in attaining food security^31^. However, the effectiveness of these strategies differs per crop and per region, and should be fully investigated before implementation.

*Agricultural indicators* correlate well with the FS_p_ values. The FS_p_ value increases in response to a heightened availability of croplands (Fig. 5ii), particularly in irrigated croplands (Fig. 5). Over the study period 2005–2015, 59–100% of the overall FS_p_ value is fulfilled by irrigated production (Table 1), which highlights the vital role of irrigated agriculture in food production in Iran. Smart long-term decisions in both irrigated and rainfed farming improves crop production per capita and smoothens the path for FS_p_ values.

Ownership over farmland also has a positive correlation to food security at a provincial scale (Fig. 5). In Iran, small-scale farmers mainly cultivate staple crops for a higher contribution in their food baskets. Whereas, large-scale farms mostly produce cash crops with a higher unit value ($ t^−1^) and a smaller contribution in Iranian’s food basket, mainly cultivated for economic gains. This is aligned with the correlation between FS_p_ values and gross income per unit of cropland ($ ha^−1^).

*Economic indicators* also correlate with FS_p_ values. FS_p_ value improves in response to gross income decrease ($ ha^−1^) (Fig. 5iii). Furthermore, gross income per unit of cropland declines when the size of cropland for each farmer increases (Fig. 5). For these farmers, agriculture is an activity catered towards nourishment rather than towards an economic gain. The stakeholders of large-scale farms are mainly commercial farmers who try to turn agriculture into an income-generating enterprise.

Small-scale farmers have key roles in food security provisions across the globe; about 80% of food security in the MENA region is supported by these farmers^32^. However, the livelihood of small-scale farmers is threatened when they unfairly compete with large-scale market-oriented farmers in national or global markets. Supporting small-scale farmers, the major producers of staple crops worldwide, and equipping them with appropriate skills enhances crop productivity and consequently, the total production of crops and diminishes unsustainable and inefficient water use in the agricultural sector. A comprehensive study conducted on 37 million ha of croplands in 57 developing countries claim that trained small-scale farmers who adopt sustainable agriculture can improve the crop yield by 79%^33^. Sustainable agriculture by small-scale farmers can terminate the unemployment rate in rural areas and may serve to motivate them in following national food production policies and contribute effectively in supplying local food demand.

*Social indicators* have a strong relationship with FS_p_ values. An increase in population density (Fig. 5iv), in particular in the proportion of urban population (Fig. 5iv), results in a considerable reduction in food security. This may arise following adverse impacts of urbanization on food security, particularly due to a reduction in the per capita cropland availability and less food production. This is accompanied by changes from small-scale farming in rural areas to market-oriented cash crop farming. Our results illustrate a positive correlation between the proportion of urban population and the gross income obtained per unit of cropland. These findings align with Overman and Venables^34^, who stated that rapid urbanization in developing countries is a driver for their economic growth and development while posing a considerable threat to several different dimensions of food security^35^. The rapid growth of urbanization in most provinces of Iran^36^ requires an urgent and long-term action driven by policy makers to meet the future demands of the nation.

The role of gender in food security is also undeniable^37,38^. Our results revealed that rural women in each province have strategic roles in satisfying provincial food security. Larger ratio of female to male in rural areas per province reflects positively on the FS_p_ rate (Fig. 5iv). While there are numerous studies which emphasize the key role of female farmers in food production and food security (e.g.^39,40^), they are rarely recognized in practice. In most developing countries, public policies and social institutes do not appreciate and support female farmers’ efforts adequately since most of the political and socio-economic determinants are extremely gendered (76); hence, female farmers continuingly face severe inequality, in terms of major development indicators, and precarity in many aspects (e.g.^40–42^). Increasing agricultural productivity therefore requires rethinking the fundamental role of women in providing food security, accompanied by structural and financial support (e.g., the inclusion of women’s empowerment components in food security programs) geared towards constructing gender-equality^37^. FAO stated that the feminization of agriculture in the Near East countries, including Iran, is less pronounced, and the number of female-headed households is considerably lower than male-headed ones^5^. Nevertheless, a large number of rural men have migrated to larger cities for higher-income jobs^40^. This is one of the reasons for the increasing number of female-headed families in recent years. More comprehensive research is required to evaluate the current state of affairs regarding gender relations and the role of female farmers in this matter.

## Conclusion

In an attempt to attain food security in water-limited countries, in this research, we adopted two food security scenarios to assess the sustainability and efficiency of crop-related blue water consumption and VW trade in Iran: (1) ICFS, in which required calorie per capita is fulfilled by individual crops, and (2) CCFS scenario, in which eating local food is promoted and the required calorie is fulfilled by the crop category. First, food security, efficiency and sustainability of blue water consumption, and VW trades of each province were determined. Then, the impact of twelve agro-economic and socio-environmental indicators on the provincial food security were tested.

The main concluding remarks of this research are:

- Crop production and trade in Iran imply inefficient and unsustainable blue water consumption under both ICFS and CCFS scenarios; this requires an effective, sustainable and long-term plan to meet the food security goal of the nation.
- The CCFS scenario (eating locally) lowers the risk of food insecurity, since more provinces bridge the gap between their food supply and demand. However, in the case of Iran, these results can be effective only when present food production policies are revisited in favor of local crop products.
- Restricting crop production in hotspot regions and adopting more efficient agriculture will help reduce inefficiency and unsustainability in crop-related water consumption which in turn results in higher levels of local food security.
- Rapid urbanization poses a great threat to provincial food security. The number of market-oriented farmers is higher in provinces with higher urban populations; this translates to a diminished contribution in provincial food production and a higher contribution in unsustainable and inefficient blue water consumption.
- Staple crops have the highest contribution in the national blue water consumption. Small-scale farmers are largely in favor of staple crops and, as such, could play a predominant role in provincial food security. This calls for action plans (e.g. provincial policies, national regulations, etc.) to support their activities.
- Rural women play a key role in fulfilling food demands; gender-equitability programs in the crop production sector are inevitable for increasing provincial food security.

Due to data limitations, our study utilized data from the period 2005–2015, as the data on provincial blue water availability was solely available for this specified time frame. As such, the results can be further complemented as newer and more detailed data becomes available. Despite these unavoidable constrains, this study still makes an important contribution to science and policy making in Iran and many other water-limited countries with similar or worsened conditions in availability or accessibility to regional or national data.

We discussed possible solutions to alleviate inefficiency and unsustainability in crop-related water consumption and trade based on agro-economic and socio-environmental indicators. Further research is required to integrate different aspects of food security, including its governance in order to assess the consequences of implementing these solutions. This encompasses investigating the feasibility of adopting these strategies in practice, and not separate to the socio-political and environmental context in which solutions must arise. Additional studies are needed to assess the impact of climate change and the readiness and capacity of the country to adapt and change.

## Materials and methods

### Case study

Facing serious water scarcity, Iran was selected as a case study for this research (see Fig. S1 in the supplementary information). Such blue water scarcity is predominantly associated with the fact that Iran is historically a water scarce country^43^. A warming climate, frequent droughts, and adverse changes in annual precipitation and surface runoff complicate water shortage problems^44^. Furthermore, political and economic sanctions hampered the technological capacity of the country which had increasing ripple effects on blue water shortages, particularly in the agricultural sector^43,45^. Previous studies showed that, over the past decades, most water challenges in Iran were man-made due to the incompatibility of national and regional development plans for blue water to become available^44,46^. In other words, blue water demand had continuously exceeded its availability leading to the overexploitation of water resources^20,22,43,47^.

Iran spans an area of about 1.64 million km^2^; divided into 30 provinces up until 2009; and classified into five climatic zones^20^. The average national population over the study period of 2005–2015 was 75.1 million people, about 29% of which living in the rural area. The provincial population density and the portion of urban population varies in the ranges of 1.2–72.3 people km^−2^ and 49–95%, respectively. In 2009, Tehran province was divided into two provinces of Tehran and Alborz, dividing Iran into 31 provinces. The larger division of these two embraces 69% of the area and 83% of the population; we called it New-Tehran in this research. New-Tehran is the capital of Iran and the most-populated province; it has about 74% of irrigated area and 71% of crop production of Tehran during 2009–2015. Since there is no data for the Alborz during 2005–2009 (this province did not exist), we carried on with the initial administrative divisions of 30 provinces for the entire study period (2005–2015). This means that what we report for Tehran province after 2009, when it inherently includes the Alborz province.

Our study period is limited to 2005–2015 due to data limitations in one of major drivers in sustainability and efficiency assessment of the agricultural sector, namely the provincial blue water availability. Although other required data was available for longer periods, a short-term record of provincial blue water availability was the largest constraint for a longer study.

Twenty seven major irrigated crops of Iran were studied; they classified into eight categories of cereals (wheat, barley, maize, and rice), vegetables (tomato and onion), pulses (bean, pea, and lentil), roots and tubers (potato), sugar crops (sugar beet and sugar cane), oil crops (cottonseed, soybean, and canola), nuts (pistachio, walnut, almond, and hazelnut), and fruits (apple, banana, date, grape, lime, lemon, tangerine, orange, and grapefruit). Over the study period (2005–2015) a total of 46.7 million t y^−1^ crops were produced within the 7.9 million ha y^−1^ irrigated lands under these crops^48^. Considering all 27 crops, arid and semi-arid regions were main crop producers of the country; arid regions by covering 51% of the total harvested area and 62% of crop production, and semi-arid regions by covering 29% of the total harvested area and 23% of crop production (see Fig. S2 in the supplementary information). Sugar crops and cereals were the main irrigated crop categories in the arid and semi-arid regions, respectively, regarding their contributions in regional production of the irrigated crops (30% and 41%, respectively).

### Food security assessment and crop export

To assess the (self-sufficient) food security level of each province (FS_P_), two scenarios were developed: Individual Crop Food Security (ICFS) and Crop Category Food Security (CCFS).

- ICFS: to feed the population of each province, the required calories per capita need to be fulfilled from each crop. FS_P_ value is less than 100%, when at minimum for one crop, the demand (required calorie from a specific crop) is higher than its local supply.

In this scenario, FS_P_ value was estimated per province using Eq. (1):

1 $$for\;ICFS\;scenario~ \to ~FS_{P} = \left( {1 - \frac{{\sum\nolimits_{{i = 1}}^{{27}} {max\left( {0,\left( {demand \cdot C_{{i,p}} - supply \cdot C_{{i,p}} } \right)} \right.} }}{{\sum\nolimits_{{i = 1}}^{{27}} {demand \cdot C_{{p,i}} } }}} \right) \times 100\%$$

where $demand \cdot C$ is per capita demand (calorie cap^−1^), $supply \cdot C$ is per capita local supply (i.e., supplied through production within the considered province, calorie cap^−1^), *i* is the number of a specific crop (*i* = 1–27), and *p* denotes the province.

A province exports a specific crop when its per capita production is higher than its per capita demand. Then the volume of annual export of each crop from each province ($CE_{{i,p}}$, t y^−1^) is estimated by multiplying per capita surplus production and province population. Per province, surplus calorie production (per capita) for each crop ($SP_{{i,p}}$, t y^−1^) is estimated under ICFS using Eq. (2):

2 $$for\;ICFS\;scenario~ \to SP_{{i,p}} = \left( {0,~\left( {supply \cdot C_{{i,p}} - demand \cdot C_{i} ,p} \right)} \right)~ \times R_{{i,p}}$$

where $R_{{i,p}}$ is a ratio for converting calorie to kilogram for crop *i* in province *p* (kg cal^−1^), and *i* is the number of a specific crop.

- CCFS: to feed the population of each province, the required calories per capita are fulfilled by each crop or a substituted crop within the crop category. In this scenario, the deficit production for a specific crop is compensated by the calorie provided by other crops in that category. For instance, wheat deficit can be compensated by any cereal. The FS_P_ value is less than 100%, when at least for one crop category, the required calorie is less than its local supply.

In this scenario, the FS_P_ value was estimated per province using Eq. (3):

3 $$for\;CCFS\;scenario~ \to ~FS_{P} = \left( {1 - \frac{{\sum\nolimits_{{j = 1}}^{8} {max\left( {0,\left( {demand \cdot C_{{j,p}} - supply \cdot C_{{j,p}} } \right)} \right.} }}{{\sum\nolimits_{{i = 1}}^{{27}} {demand \cdot C_{j} ,p} }}} \right) \times 100\%$$

where *j* is the number of crop category (*j* = 1–8), *p* denotes the province. Per province, surplus calorie production (per capita) for each crop ($SP_{{i,p}}$, t y^−1^) is estimated the CCFS scenarios using Eq. (4):

4 $$for\;ICFS\;scenario~ \to SP_{{i,p}} = \left( {0,~\left( {supply \cdot C_{{i,p}} - demand \cdot C_{i} ,p} \right)} \right)~ \times R_{{i,p}}$$

5 $$for\;CCFS\;scenario \to SP_{{i,p}} = \frac{{supply \cdot C_{{i,p}} }}{{supply \cdot C_{{j,p}} }} \times \left( {max\left( {0,~\left( {supply \cdot C_{{j,p}} - demand \cdot C_{{j,p}} } \right)} \right) \times R_{{i,p}} } \right)$$

where *j* is the number of the crop category in which crop *i* is located.

Due to lack of data on inter-provincial crop trade, this study does not incorporate real conditions, as an individual scenario. This modeling exercise is limited with the best knowledge and data we have in the time of publication. We assessed possible pathways under certain assumptions to explore the extent and magnitude of their impacts in the case of implementation. Our findings inform decision-makers about their options and how likely each option affects agro-economic and socio-environmental settings from a water and food security perspective on provincial or national scales.

Since our main focus is on assessing internal trades and introducing opportunities to optimize the utilization of internal water resources in favor of food security in a sustainable manner, external trades were excluded from our assessments.

### Sustainability and efficiency assessment

The sustainability of blue water consumption or VW trade is assessed based on two key indices of (1) water stress index (WSI), developed by Pfister et al.^49^, and (2) blue water scarcity (BWS), developed by Hoekstra et al.^7,50^. The WSI combines the utilization of water resources with their environmental impacts to qualify water resource sustainability. It ranges from 0 to 100%, indicating the percentage of freshwater use which was consumed at the expense of depriving the other users from freshwater. The applicability of this index was demonstrated through a global life cycle assessment of cotton production by Pfister et al.^49^. The BWS is defined as the ratio of total blue water consumption (i.e., total blue WF value) to the local blue water availability. The BWS = 1 indicates that the sustainable available blue water has been fully consumed. A BWS > 1 denotes that blue water has been consumed at the expense of violating environmental flow requirements; hence, the portion of the consumptive blue WF value beyond its sustainable level is labelled “unsustainable consumption”. We applied BWS in the current research to assess the sustainability of water consumption and VW trade.

To assess the efficiency of blue water consumption and VW trade, we applied the concept of WF benchmarking. Earlier researchers indicated that WF benchmarking is a promising strategy for reducing the inefficient blue water consumption through the crop production process^22,51,52^.

#### WF of crop production

WF calculations were done per crop, per province and per year. Crop’s daily ET was simulated by the AquaCrop model^53^. The following daily soil water balance is simulated by the model for the rooting zone using Eq. (6).

6 $$S_{t} = S_{{\left[ {t - 1} \right]}} + P_{{\left[ t \right]}} + I_{{\left[ t \right]}} + CR_{{\left[ t \right]}} - ET_{{\left[ t \right]}} - RO_{{\left[ t \right]}} - DP_{{\left[ t \right]}}$$

where $S_{{\left[ t \right]}}$ and $S_{{\left[ {t - 1} \right]}}$ are the soil water content at the end of day *t* and *t* − 1, respectively, $P~$ is precipitation, $I$ is irrigation, $CR$ is capillary rise, $ET$ is evapotranspiration, $RO$ is surface runoff, and $DP$ is deep percolation on day *t*. All parameters are in mm day^−1^.

Green and blue WF values were estimated by dividing the seasonal green and blue evapotranspiration (*ET*, m^3^ ha^−1^) by crop yield (t ha^−1^), respectively^7^. I and CR were considered blue water; and P was considered green water. CR was divided into green and blue CR based on the ratio of P and I into P + I, respectively. *DP* and *ET* were divided into green and blue components based on the fraction of *S*_*green*_ and *S*_*blue*_ in total *S* at the end of the previous day. Finally, soil green $\left( {S_{{green}} } \right)$ and blue $\left( {S_{{blue}} } \right)$ water contents were estimated as follows^54^:

7 $$S_{{green\left[ t \right]}} = S_{{green\left[ {t - 1} \right]}} + P_{{\left[ t \right]}} + RO_{{\left[ t \right]}} \times \frac{{P_{{\left[ t \right]}} }}{{P_{{\left[ t \right]}} + I_{{\left[ t \right]}} }} - \left( {DP_{{\left[ t \right]}} + ET_{{\left[ t \right]}} } \right) \times \frac{{S_{{green\left[ {t - 1} \right]}} }}{{S_{{\left[ {t - 1} \right]}} }}$$

8 $$S_{{blue\left[ t \right]}} = S_{{blue\left[ {t - 1} \right]}} + I_{{\left[ t \right]}} + RO_{{\left[ t \right]}} \times \frac{{I_{{\left[ t \right]}} }}{{P_{{\left[ t \right]}} + I_{{\left[ t \right]}} }} - \left( {DP_{{\left[ t \right]}} + ET_{{\left[ t \right]}} } \right) \times \frac{{S_{{blue\left[ {t - 1} \right]}} }}{{S_{{\left[ {t - 1} \right]}} }}$$

Seasonal green and blue ET were then calculated by aggregating the daily green and blue ET over the whole growing period of the crop.

To take the variation of per capita water demand across provinces into account, the total blue water consumption of a specific crop ($total \cdot blue~WF_{{i,p}}$, m^3^ y^−1^) for each province was calculated as follows by Eq. (9).

9 $$total \cdot blue\;WF_{{i,p}} = blue\;WF_{{i,p}} \times TP~_{{i,p}}$$

where $blue\;WF_{{i,p}}$ is blue WF value of crop *i* in province *p* (m^3^ t^−1^), and $TP_{{i,p}}$ is the provincial production volume of crop *i* (t y^−1^). Summing up all crops grown in a specific province, provincial blue water consumption of crop production $\left( {total \cdot blue\;WF_{p} } \right)$ was then estimated.

#### Sustainability and efficiency of blue WFs

*Sustainability of blue WFs*—Provincial blue water scarcity (BWS) is calculated by dividing $total \cdot blue\;WF_{p}$ by local blue water availability in a province^50^. Per province, local water availability was calculated as local natural runoff minus environmental flow requirement^7^. The latter was assumed as 80% of the local natural runoff following Richter et al.^55^. BWS ≤ 1 implies that part of the local water availability is still left for the environmental flow; hence, $total \cdot blue\;WF_{p}$ is sustainable. When BWS > 1, then part of the $total \cdot blue\;WF_{p}$ will be unsustainable and could be calculated as follows:

10 $$\left( {total \cdot blue\;WF_{p} } \right)_{{unsustainable}} = total \cdot blue\;WF_{p} - BWA_{p}$$

where $BWA_{p}$ is local water availability in province *p* (m^3^ y^−1^).

*Efficiency of blue WFs*—To assess the efficiency of WFs related to crop production, the blue WF benchmarks levels were considered as references. If the provincial blue WF value of a specific crop is less than its blue WF benchmark, that crop’s production will be efficient^18^. Here, the climate-specific blue WF benchmarks calculated by Karandish et al.^22^ for the 27 crops grown in the study area were considered in the efficiency assessment. Per crop and per province, the inefficient $total \cdot blue\;WF_{{i,p}}$ ($\left( {total \cdot blue\;WF_{{i,p}} } \right)_{{inefficient}}$, m^3^ y^−1^) were then calculated using Eq. (11).

11 $$\left( {total \cdot blue\;WF_{{i,p}} } \right)_{{inefficient}} = \left( {blue\;WF_{{i,p}} - BM \cdot blue\;WF_{{i,p}} } \right) \times TP_{{i,p}}$$

where $BM \cdot blue\;WF_{{i,p}}$ is the blue WF benchmark for crop *i* in province *p*. Summing up all crops grown in the considered province, the total inefficient blue WF values related to crop production were calculated.

#### Sustainability and efficiency of inter-provincial VW export

Per province, the total gross inter-provincial blue *VW* export ($gross\;blue\;VWE_{p}$, m^3^ y^−1^) related to the 27 selected crops was estimated as follows:

12 $$gross\;blue\;VWE_{p} = \mathop \sum \limits_{{i = 1}}^{{27}} CE_{{i,p}} \times blue\;WF_{{i,p}}$$

where $CE_{{i,p}}$ is the volume of annual export of crop *i* from province *p* (t y^−1^), and $blue\;WF_{{i,p}}$ is the provincial blue *WF* value per unit production of crop *i* (m^3^ t^−1^). Having the $gross\;VWE_{p}$, the unsustainable and inefficient $gross\;VWE_{p}$ (i.e., $unsustainable\;gross\;VWE_{p}$ and $inefficient\;gross\;VWE_{p}$, respectively, expressed in m^3^ y^−1^) was determined as follows.

13 $$unsustainable\;gross\;VWE_{p} = \mathop \sum \limits_{{i = 1}}^{{27}} \frac{{\left( {abs \cdot blue\;WF_{{i,p}} } \right)_{{unsustainable}} }}{{abs \cdot blue\;WF_{{i,p}} }} \times CE_{{i,p}} \times blue\;WF_{{i,p}}$$

14 $$Inefficient\;gross\;VWE_{p} = \mathop \sum \limits_{{i = 1}}^{{27}} \frac{{\left( {abs \cdot blue\;WF_{{i,p}} } \right)_{{inefficient}} }}{{abs \cdot blue\;WF_{{i,p}} }} \times CE_{{i,p}} \times blue\;WF_{{i,p}}$$

### Normalizing the results

Since different provinces have different areas and resources, we provided normalized values per provincial crop export, blue WF value and blue VW export (i.e., total, unsustainable, and inefficient) to make them comparable. In this regard, we divided the given results by provincial harvested area. For instance, for a specific province, the unit unsustainable blue WF value (m^3^ ha^−1^) was estimated by dividing the unsustainable blue WF value (m^3^) by the harvested area of 27 crops in that province (ha^−1^). The same procedure was adopted for other indices.

### Agricultural, economic, environmental and social indicators

We assessed the relationship between provincial food security level, agro-economic and socio-environmental factors. Considering limitations in data availability and the available literature, we shortlisted key agro-economic and socio-environmental indicators and studied the relationship between food security and these indicators at a province scale (see Table S1 in the supplementary information). These indicators include:

- Agricultural indicators: cropland availability, irrigated land availability, and cropland authorship.
- Social indicators: population density, urban/rural portion, and female/male portion.
- Economic indicators: gross income from cropland, gross income per capita, and gross income per rural capita.
- Environmental indicators: water availability per unit of cropland, per capita water availability, and BWS.

### Data

The required data was obtained per crop, per province and per year over the study period (2005–2015). Meteorological data was obtained from 52 synoptic stations spread over the study area^56^. The provincial averages were then acquired from local and national organizations and fed into the AquaCrop model. Soil features were extracted from the 5 × 5 arc min raster maps provided by Batjes^57^ and were then converted into provincial average values. Provincial natural runoff received from national reports were provided by the Water Resource Management Company (WRM)^58^. All agricultural data including cropping calendars (i.e., the growing periods), agricultural practices and water management were supplied by the Ministry of Agriculture Jihad of Iran (IMAJ)^48^ which were compiled from provincial reports. Producer prices for each crop in each province were procured from the IMAJ^48^. Provincial per capita crop demands were derived from data published by the Statistical Center of Iran^59^. More details are available in Table S2 in the supplementary information.

## Supplementary Information

Supplementary Information 1.
