# Supplementary material for: Agro-economic and socio-environmental assessments of food and virtual water trades of Iran
Source: Sci Rep. 2021 Jul 22;11:15022. doi: 10.1038/s41598-021-93928-9 (PMC8298399; doi:10.1038/s41598-021-93928-9)

**Agro-economic and socio-environmental assessments of food and virtual water trades of Iran**

Fatemeh Karandish^1*^, Hamideh Nouri^2^, Marcela Brugnach^3,4^

^1^ Multidisciplinary Water Management, Faculty of Engineering Technology, University of Twente, P.O. Box 217, 7500 AE Enschede, Netherlands

^2^ Division of Agronomy, University of Göttingen, Von-Siebold-Strasse 8, 37075, Göttingen, Germany

^3^ Basque Centre for Climate Change, Scientific Campus of the University of the Basque Country, Leioa 48940, Spain

^4^ Basque Foundation for Science, Ikerbasque, Bilbao, Spain

* Corresponding author, Email: [f.karandish@utwente.nl](mailto:f.karandish@utwente.nl) & Karandish_h@yahoo.com

**Supplementary information**

1. **Supplementary Tables**

**Table S1.** The list of selected agricultural, economic, environmental, and social indicators for correlation analysis.

**Table S2.** An overview of the type and source of data used in the current research.

**Table S3.** The national average of per capita food and calorie demand in the study area

1. **Supplementary Figures**

**Fig. S1. S**tudy area, map of Iran (left), population density and portion of urban population (middle), and synoptic stations and climatic zones of Iran (right). Numbers in the middle map denotes the ratio of the population of the province to the total population of Iran.

**Fig. S2.** Provincial distribution of (a and d) the total and (b and e) unit croplands and production for all 27 crops in the study area,; (c) the contribution of different crop categories in total cropland and (f) production per climatic zone as average values over the study period (2005-2015). Per province, unit cropland was estimated by dividing the area of croplands by the province area, and unit production was divided by the total production of that province by its total cropland.

**Fig. S3.** Spatial distribution of the 10-year average of (a) natural runoff, (b) environmental flow requirements (EFR), (c) and blue water availability (BWA) in Iran over the study period (2005-2015)

**Table S1.** The list of selected agricultural, economic, environmental, and social indicators for correlation analysis


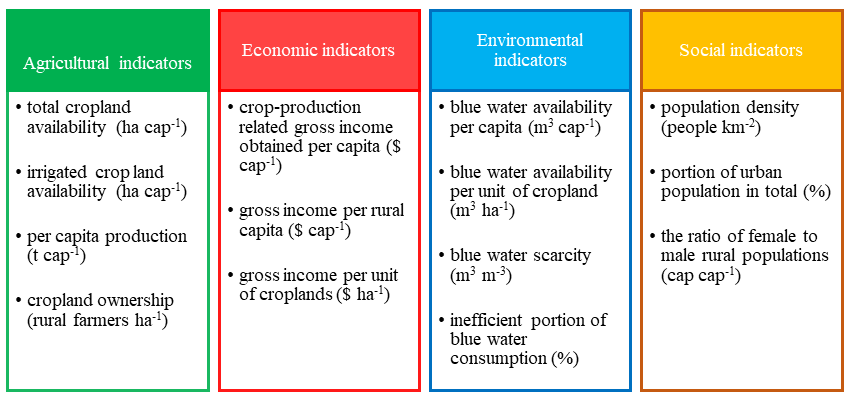


**Table S2.** An overview of the type and source of data used in the current research

| Type of data | Descriptions | spatial resolution | Source |
| --- | --- | --- | --- |
| Meteorological data | Weather data (Tmin, Tmax, RH, WS, n) | 52 synoptic stations | IRIMO (2020) |
|  | Climatic zones | province | Karandish and Hoekstra (2017) |
| Agricultural data | harvested area, crop yield and total production per crop, (i.e., with distinguishing between irrigated and rainfed types), irrigation information, fertilizations, cropping calendars | province | IMAJ (2020) |
| Food demand | Data on per capita consumption for each crop expressed in kg cap^-1^ and kcal cap^-1^ | National scale | FAO (2020) |
| Social data | Total population, portion of urban and rural population, and female to male ratio | province | ISC (2020) |
| Economic data | producer price ($ t-1) per crop | national average | FAO (2020) |
| Environmental data | blue water availability, | province | WRM (2020) |
| Climate-specific WF benchmark levels | green and blue WF benchmark levels per individual crops | climatic zones | Karandish et al. (2018) |
| Soil data | Soil texture, and total water holding capacity | 5×5 arc min | Batjes (2012) |
|  | Soil hydraulic properties including PWP, FX, TS, and KS | Not applicable | Steduto et al. (2009) |

**Table S3.** The national average of per capita food and calorie demand in the study area

| Crops | Food demand (kg cap^-1^) | Calori demand (1000 Kcal cap^-1^) |
| --- | --- | --- |
| Wheat | 153.4 | 166664.5 |
| Barley | 0.7 | 799.4 |
| Rice | 31.0 | 37169.8 |
| Maize | 2.7 | 3197.4 |
| Onion | 21.5 | 3330.6 |
| Tomato | 55.7 | 3863.5 |
| Potato | 45.7 | 12523.2 |
| Soybean | 14.4 | 62.8 |
| Cotton | 1.8 | 0.4 |
| Canola | 4.7 | 14.2 |
| Sugarbeet | 55.2 | 41.7 |
| Sugarcane | 76.3 | 57.6 |
| Nuts* | 12.1 | 12922.8 |
| Bean | 3.9 | 4929.3 |
| Lentil | 5.9 | 20.8 |
| Apple | 13.7 | 2398.1 |
| Banana | 10.2 | 2264.8 |
| Citrus** | 37.8 | 2531.3 |
| Date | 10.9 | 6261.6 |
| Grape | 19.7 | 5195.8 |
| * Nuts include pistachio, walnut, almond, and hazelnut | | |
| ** Citrus includes lime, lemon, tangerine, orange, and grapefruit | | |

**Fig. S1. S**tudy area, map of Iran (left), population density and portion of urban population (middle), and synoptic stations and climatic zones of Iran (right). Numbers in the middle map denotes the ratio of the population of the province to the total population of Iran. (i.e., This Figure is created in the environment of ArcMap-GIS version 10.7)


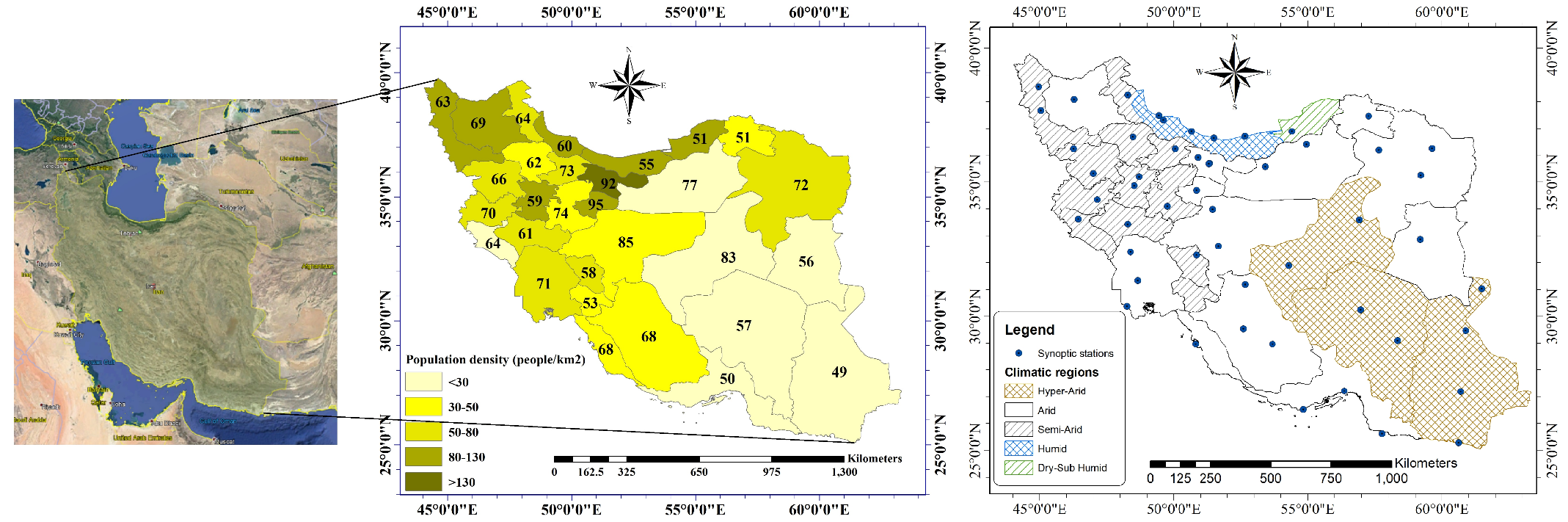


**Fig. S2.** Provincial distribution of (a and d) the total and (b and e) unit croplands and production for all 27 crops in the study area,; (c) the contribution of different crop categories in total cropland and (f) production per climatic zone as average values over the study period (2005-2015). Per province, unit cropland was estimated by dividing the area of croplands by the province area, and unit production was divided by the total production of that province by its total cropland. (i.e., This Figure is created in the environment of ArcMap-GIS version 10.7)

**
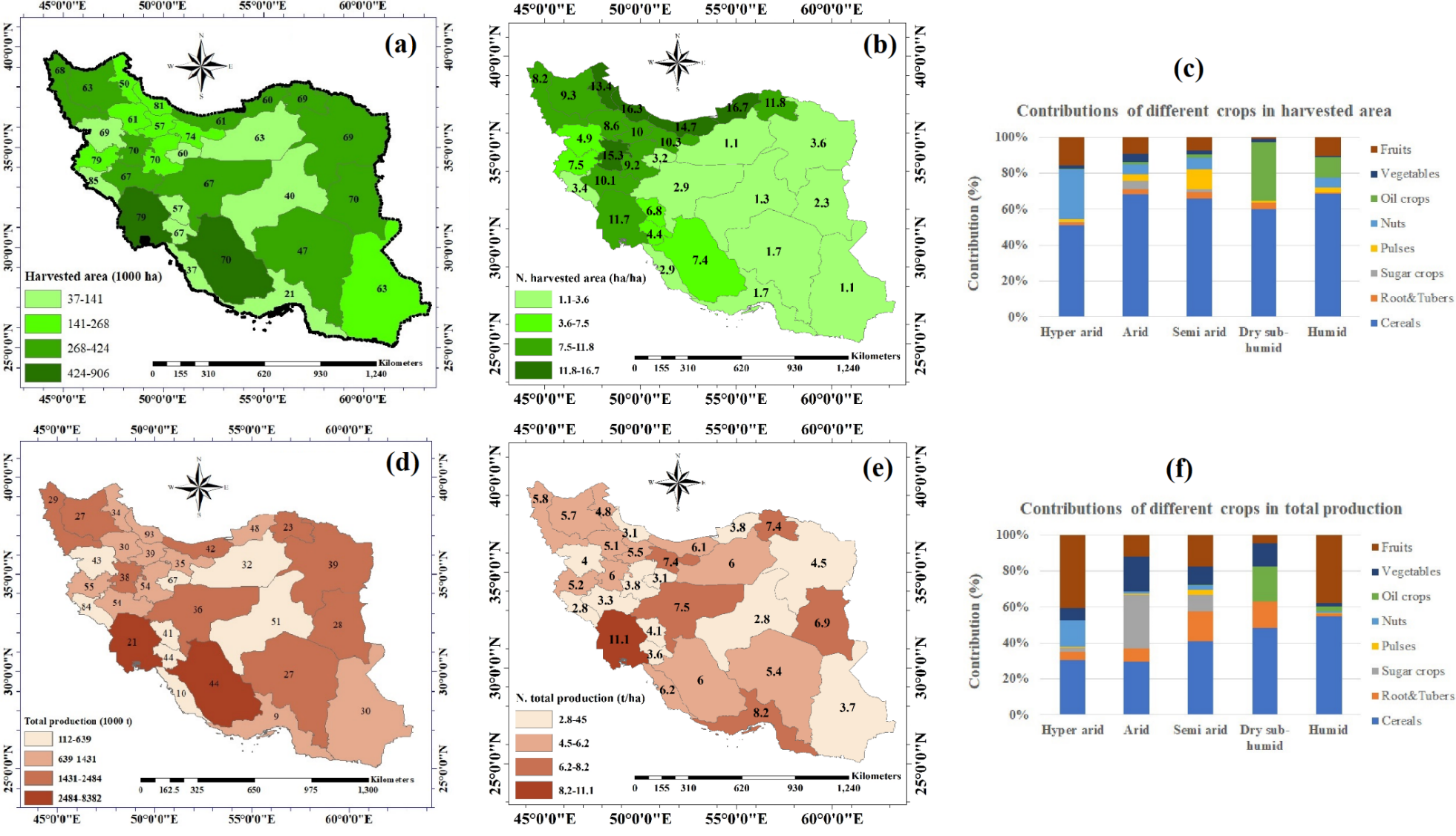
**

**Fig. S3.** Spatial distribution of the 10-year average of (a) natural runoff, (b) environmental flow requirements (EFR), (c) and blue water availability (BWA) in Iran over the study period (2005-2015). (i.e., This Figure is created in the environment of ArcMap-GIS version 10.7)


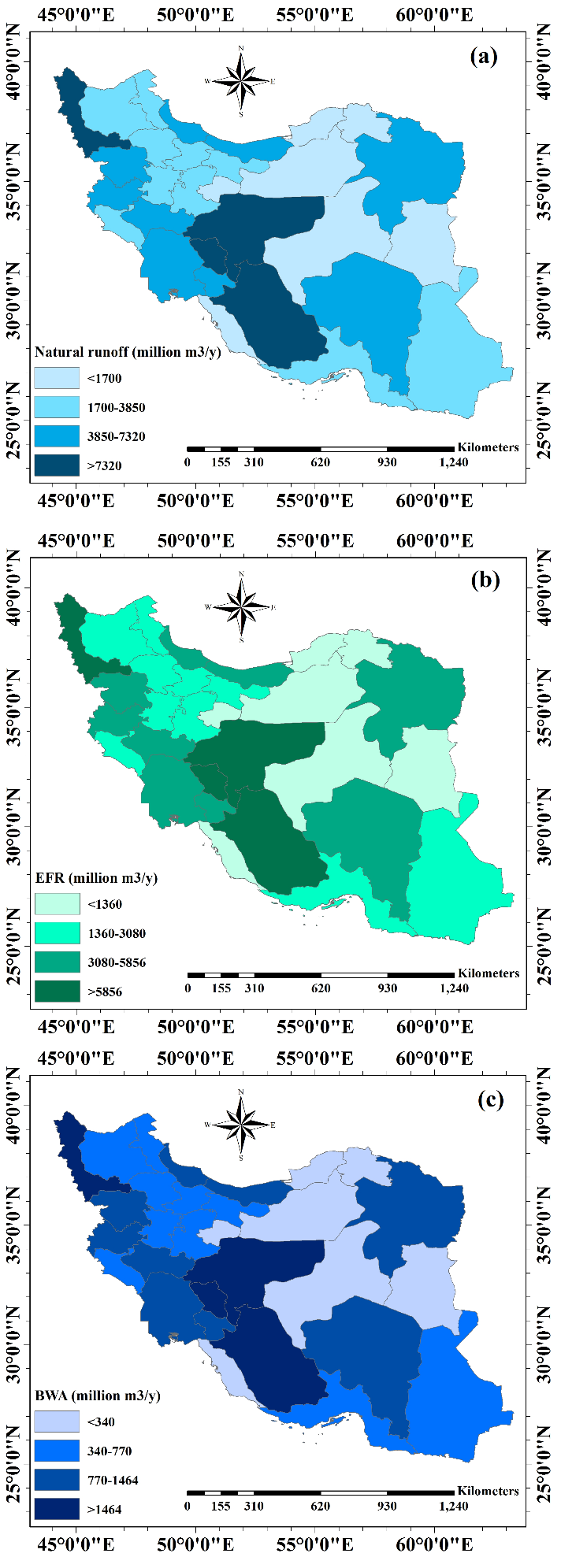

Supplement: Supplementary file 1 — Supplementary Information 1. [file 41598_2021_93928_MOESM1_ESM.docx]
